# The clinical outcomes of reni-angiotensin system inhibitors for patients after transcatheter aortic valve replacement: A systematic review and meta-analysis

**DOI:** 10.3389/fcvm.2022.963731

**Published:** 2022-08-11

**Authors:** Shuai Wang, Xiaoxiao Lin, Yihong Guan, Jinyu Huang

**Affiliations:** ^1^Department of Translation Medicine Center, Affiliated Hangzhou First People's Hospital, Zhejiang University School of Medicine, Hangzhou, China; ^2^Department of Cardiology, Affiliated Hangzhou First People's Hospital, Zhejiang University School of Medicine, Hangzhou, China; ^3^The Fourth School of Clinical Medicine, Zhejiang Chinese Medical University, Hangzhou, China

**Keywords:** transcatheter aortic valve replacement (TAVR), clinical outcomes, reni-angiotensin system (RAS) inhibitors, all-cause mortality, a systematic review and meta-analysis

## Abstract

**Aims:**

The objective of our systematic reviews and meta-analysis is to evaluate the clinical outcomes of RAS inhibitors for patients after TAVR.

**Methods and results:**

We performed a comprehensive search for Embase, Pubmed, and Cochrane databases from inception to May 1, 2022. The analysis of all outcomes was performed using the random-effects model. In total, 7 articles with a total of 32,585 patients (RAS inhibitor, *N* = 14,871; Controls, *N* = 17,714) were included in our study. There was a significantly lower rates of all-cause mortality (RR = 0.76, 95%Cl = 0.68 to 0.86, *P* < 0.01), cardiovascular death (RR = 0.66, 95%Cl = 0.59–0.74, *P* < 0.01) and HF readmission (RR = 0.87, 95%Cl = 0.80–0.94, *P* < 0.01) in patients with RAS inhibitors compared with controls. Patients with RAS inhibitors also had lower rates of all-cause mortality (RR = 0.82, 95%Cl = 0.76–0.89, *P* < 0.01) and cardiovascular death (RR = 0.73, 95%Cl, 0.62–0.85, *P* < 0.01) after propensity matching.

**Conclusions:**

In conclusion, our systematic reviews and meta-analysis demonstrated that RAS inhibitors could improve the clinical outcomes for patients after TAVR. Further large and high-quality trials should be conducted to support the use of RAS inhibitors for patients after TAVR.

## Introduction

Aortic stenosis (AS) was a common valve disease and one of the major cardiovascular morbidities in the aging population, which was estimated that more than 5% of adults who were older than 75 years were affected by AS (1). Overload chronic pressure caused by aortic stenosis could promote left ventricular remodeling through abnormalities of the collagen network and muscle fiber hypertrophy, then increase the risk of heart failure (HF) and result in diastolic dysfunction (1). For patients with AS, Transcatheter aortic valve replacement (TAVR) was proven to be effective in the past decade (2–4).

Reni-angiotensin system (RAS) inhibitors could modulate regression of myocardial hypertrophy and adverse left ventricular remodeling, which may result in clinical improvements in patients after TAVR (5). Several studies evaluated the association between RAS inhibitors and the clinical outcomes of patients after TAVR. For example, Ledwoch et al. evaluated the dose of RAS inhibitors on clinical outcomes and demonstrated that increasing doses of RAS inhibitor could improve the 3-year survival in patients after TAVR (6). A recent study showed the benefits between improved survival and RAS inhibitors may be dose-dependent and particularly evident in high-risk patients (7). However, these findings from these studies were not well summarized and analyzed. We conducted the systematic reviews and meta-analysis to evaluate the clinical outcomes of RAS inhibitors for patients after TAVR.

## Methods

### Search strategy

Our study was conducted according to Cochrane Collaboration guidelines and PRISMA criteria (8, 9). We performed a comprehensive search for Embase, Pubmed, and Cochrane databases from inception to May 1, 2022. The following terms were used: (RAS OR ACEI OR “angiotensin-converting enzyme inhibitor” OR “Renin Angiotensin System” OR “RAS inhibitor” OR “RAS inhibitors” OR “RAS blockade” OR “RAS blockades” OR ARB OR “angiotensin receptor blocker” OR “Renin-Angiotensin System Inhibition” OR “reni-angiotensin system inhibitors” OR “reni-angiotensin system inhibitor” OR RASI) AND (TAVI OR TAVR OR “transcatheter aortic valve replacement” OR “transcatheter aortic valve implantation”).

### Inclusion criteria

According to the PICOS principle, we made the inclusion criteria as follows: (P) Patients: patients after TAVR. (I) Interventions: RAS inhibitor. (C) Control: without RAS inhibitor. (O) Outcomes: the primary outcome was all-cause mortality, and the second outcomes were cardiovascular death and readmission due to HF. (S) Study: clinical studies including RCTs and non-RCTs. After removal of duplicates, editorials, comments, letters to the editor, case reports, conference abstracts, and supplements, the eligible studies were identified and selected.

### Data extraction and quality appraisal

Two reviewers (Wang and Lin) independently conducted data extraction with a form containing the first author's name and publication year, sample size in RASI and No RASI groups, location, patient enrollment periods, main findings, and treatment periods, with discrepancies adjudicated by the third reviewer (Huang). For the assessment of risk of bias in each study, ROBINS-I tool of seven domains was used (10).

### Data analysis

The analysis was conducted by RevMan software, version 5.4.1 and Stata Software, Version 17.0 (Stata Corporation, College Station, TX, USA). The random-effects model was used for all outcomes. The relative risk (RR) was adopted for dichotomous outcomes. The *I*^2^ was used to assess heterogeneity, and the heterogeneity was considered moderate when the *I*^2^ index was 25%-75%, high when the *I*^2^ index was above 75%, and low when the *I*^2^ index was below 25% (11). For the primary outcome, the subgroup analyses were conducted according to: (1) the time of follow-up: 1 year, 2 years, and 3 years (2) The dose of RAS inhibitors: <50% dose and ≥50% dose. The meta-regression analyses for the effect of presence/absence of RAS on all-cause mortality were conducted according to age, sex and baseline LVEF.

## Results

### Study selection

**Figure 1:**
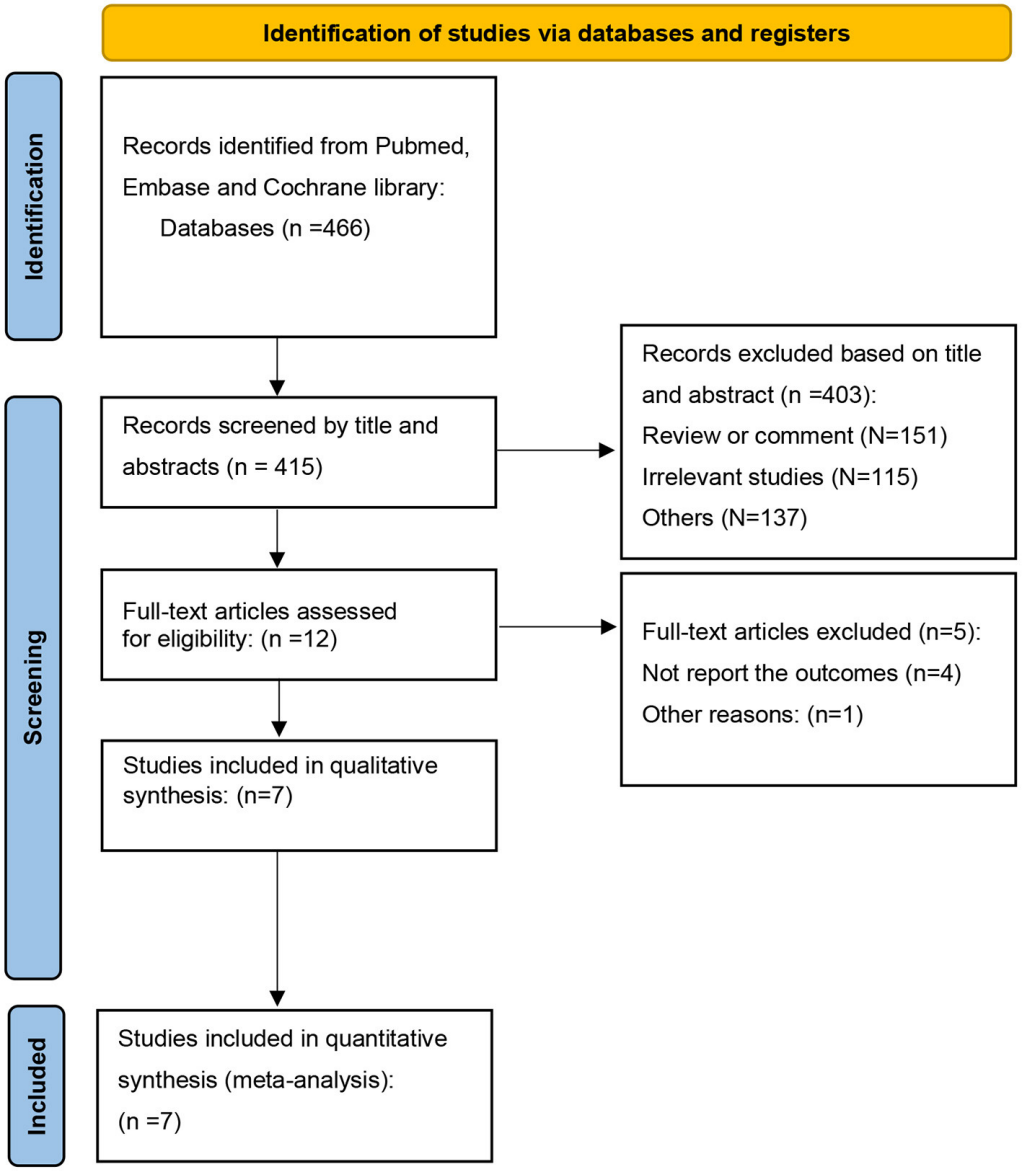
PRISMA search strategy.

A total of 466 articles were identified, after excluding duplications, 415 articles were screened by title and abstracts. 12 full-text articles were reviewed, and 5 articles were removed (12–16). Altogether, 7 articles (6, 7, 17–21) were included, with a total of 32,585 patients (RAS inhibitor, *N* = 14,871; Controls, *N* = 17,714). The full search process is shown in Figure 1. The study was reported in accordance with the PRISMA checklist (Supplementary Table S1). According to the ROBINS-I tool, all the studies were found to be low or moderate risk of bias. The assessment of risk of bias with ROBINS-I tool was summarized in Supplementary Table S2.

### Characteristics of studies and patients

For 7 included studies, the sample size ranged from 323 to 21312. The patient enrollment period ranged from 2007 to 2020. There were five retrospective studies and two prospective studies, and four studies were with propensity score-matched cohort analysis. The periods of follow-up ranged from 1 to 3 years (Table 1). For included patients with and without RAS inhibitors, the mean age was similar. The percentages of LVEF were also similar, and the characteristics of patients was shown in Table 2. The types of the RAS inhibitors were mainly ACEI and ARB. The RAS inhibitors per study were summarized in Supplementary Table S3.

**Table 1:** The Characteristics of included studies.

**Study, year**	**RASi**	**NO RASI**	**Country**	**Design**	**Multicenter**	**Periods**	**Main findings**	**Follow up, years**
Chen, (17)	1,736	2,243	US,Canada	Retrospective, PSM	Yes	2011.03–2018.08	Treatment with RAS inhibitors at baseline independently associated with a lower risk of 2-year all cause and cardiovascular mortality independently.	2
Inohara, (18)	8,468	12,844	US	Retrospective, PSM	Yes	2014.07–2016.01	Patients with RAS inhibitors were significantly associated with a lower risk of mortality and heart failure readmission.	1
Ledwoch, (6)	98	225	Germany	Prospective	No	2015.01–2019.09	The impact of RAS blockade treatment on clinical outcome after TAVR was dose dependent.	3
Ochiai, (21)	371	189	Japan	Prospective, PSM	Yes	2013.10–2016.04	RAS inhibitor therapy was associated with reduced all-cause mortality and greater lV mass index regression.	2
Rodriguez-Gabella, (20)	1,622	1,163	Spain	Retrospective, PSM	Yes	2007.08–2017.08	Post-TAVR RAS inhibitors were associated with lower cardiac mortality at 3-year follow-up.	3
Fischer-Rasokat, (7)	2,227	635	Germany	Retrospective	No	2011.01–2020.12	The improved survival during follow-up is particularly evident in high-risk patients and may be dose dependent.	3
Kaewkes, (19)	349	415	US	Retrospective	No	2013.01–2017.11	Patients treated with RAS inhibitors were associated with lower all-cause mortality and HHF at 2-year.	2

**Table 2:** The Characteristics of included patients.

	**Chen, (** **17** **)**		**Inohara, (** **18** **)**		**Ledwoch, (** **6** **)**		**Ochiai, (** **21** **)**	
**Variable**	**RASi** **(*****N*** **= 1,736)**	**No RASi** **(*****N*** **= 2,243)**	**RASi** **(*****N*** **= 8,468)**	**No RASi** **(*****N*** **= 12,844)**	**RASi** **(*****N*** **= 225)**	**No RASi** **(*****N*** **= 98)**	**RASi** **(*****N*** **= 371)**	**No RASiṟeak (*****N*** **= 189)**
Age (yrs)	81.7 ± 7.2	82.9 ± 7.7	82.3 ± 6.8	82.9 ± 6.9	80.7 ± 6.6	79.8 ± 9.3	84.2 ± 5.0	84.8 ± 5.0
Female	681 (39.2%)	932 (41.6%)	3,983 (47%)	6,087 (47.4%)	99 (44%)	45 (46%)	253 (68.2%)	124 (65.6%)
Hypertension	1,672 (96.3%)	2,012 (89.7%)	7,950 (93.9%)	11,289 (87.9%)	212 (94.2%)	77 (81%)	311 (83.8%)	116 (61.4%)
Diabetes mellitus	704 (40.6%)	729 (32.5%)	3,371 (39.8%)	4,377 (34.1%)	58 (25.8%)	18 (19%)	103 (27.8)	46 (24.3%)
Previous CABG	628 (36.2%)	596 (26.6%)	2,515 (29.7%)	3,251 (25.3%)	11 (4.9%)	10 (10%)	28 (7.5%)	11 (5.8%)
Chronic renal failure	125 (7.2%)	219 (9.8%)	3,834 (45.3%)	6,763 (52.7%)	112 (49.8%)	59 (63%)	246 (66.3%)	108 (57.1)
LVEF %	54.5 ± 13.7	54.9 ± 13.3	51.1 ± 12.1	52.6 ± 10.8	52.4 ± 10.1	51.7 ± 11.2	62.9 ± 13.1	63.3 ± 11.9
Mean gradient (mmHg)	44.7 ± 13.0	44.5 ± 13.5	NA	NA	42.4 ± 16.6	42 ± 16	50.8 ± 18.4	50.6 ± 16.8
STS-PROM score (%)	7.2 ± 3.8	7.7 ± 4.2	7.4 ± 5.0	8.3 ± 6.1	NA	NA	7.0 (4.8–9.5)	7.0 (5.0–9.4)
**Approach**
Transfemoral	1,357 (78.2%)	1,740 (77.6%)	NA	NA	196 (87.1%)	79 (80.6%)	295 (79.5%)	162 (85.7%)
Non-Transfemoral	379 (21.8%)	503 (22.4%)	NA	NA	29 (12.9)	19 (19.4%)	76 (20.5%)	27 (15.4%)
**Valve type**
Balloon-expandable valve	1,736 (100%)	2,243 (100%)	NA	NA	178 (79.1%)	83 (84.7%)	364 (98.1%)	188 (99.5%)
Self-expandable system	0	0	NA	NA	47 (20.9%)	15 (15.3%)	7 (1.9%)	1 (0.5%)
	**Rodriguez-Gabella, (** **20** **)**		**Fischer-Rasokat, (** **7** **)**		**Kaewkes, (** **19** **)**	
**Variable**	**RASi** **(*****N*** **= 1,622)**	**No RASi** **(*****N*** **= 1,163)**	**RASi** **(*****N*** **= 2,227)**	**No RASi** **(*****N*** **= 635)**	**RASi** **(*****N*** **= 349)**	**No RASi** **(*****N*** **= 415)**
Age (yrs)	80.8 ± 7.01	80.7 ± 7.18	82.0 (78.7–85.0)	82.0 (78.2–85.6)	81.4 ± 7.7	82.9 ± 8.7
Female	890 (54.9%)	617 (53.1%)	1,162 (52.2%)	336 (52.9%)	150 (43%)	164 (40%)
Hypertension	1,388 (85.6)	869 (74.8)	2,080 (93.4%)	521 (82.0%)	327 (94%)	359 (86%)
Diabetes mellitus	591 (36.4%)	368 (31.7%)	732 (32.9%)	207 (32.6%)	119 (34%)	100 (24%)
Previous CABG	140 (9.1%)	82 (8.5%)	NA	NA	61 (18%)	53 (13%)
Chronic renal failure	NA	NA	NA	NA	262 (75%)	303 (73%)
LVEF %	57.4 ± 13.9	58.9 ± 13.4	65 (55–65)	65 (55–65)	59.3 ± 14.4	58.7 ± 23.7
Mean gradient (mmHg)	47.3 ± 15.7	48.9 ± 16.6	42 (33–52)	43 (32–51)	45.0 ± 13.1	44.8 ± 14.6
STS-PROM score (%)	5.1 (3.4–7.5)	5.0 (3.5–8.0)	NA	NA	4.5 (3.0–6.6)	5.0 (3.3–7.9)
**Approach**						
Transfemoral	1,518 (93.6%)	1,040 (89.5%)	NA	NA	339 (97.1%)	395 (95.1%)
Non-transfemoral	41 (2.5%)	123 (10.6%)	NA	NA	10 (2.9%)	20 (4.9%)
**Valve type**						
Balloon-expandable valve	413 (25.5%)	334 (28.8%)	804 (36.1%)	254 (40.0%)	300 (86%)	349 (84%)
Self-expandable system	1,209 (74.5%)	829 (71.3%)	1,423 (63.9%)	381 (60%)	49 (14%)	66 (16%)

Values are expressed as events (percentages), mean ± standard deviation or median(IQR).

LVEF, left ventricular ejection fraction; CABG, coronary artery bypass grafting; RASi, renin- angiotensin system inhibitor; STS-PROM score, Society of Thoracic Surgeons Predicted Risk of Mortality.

### Outcomes

#### Primary outcomes

**Figure 2:**
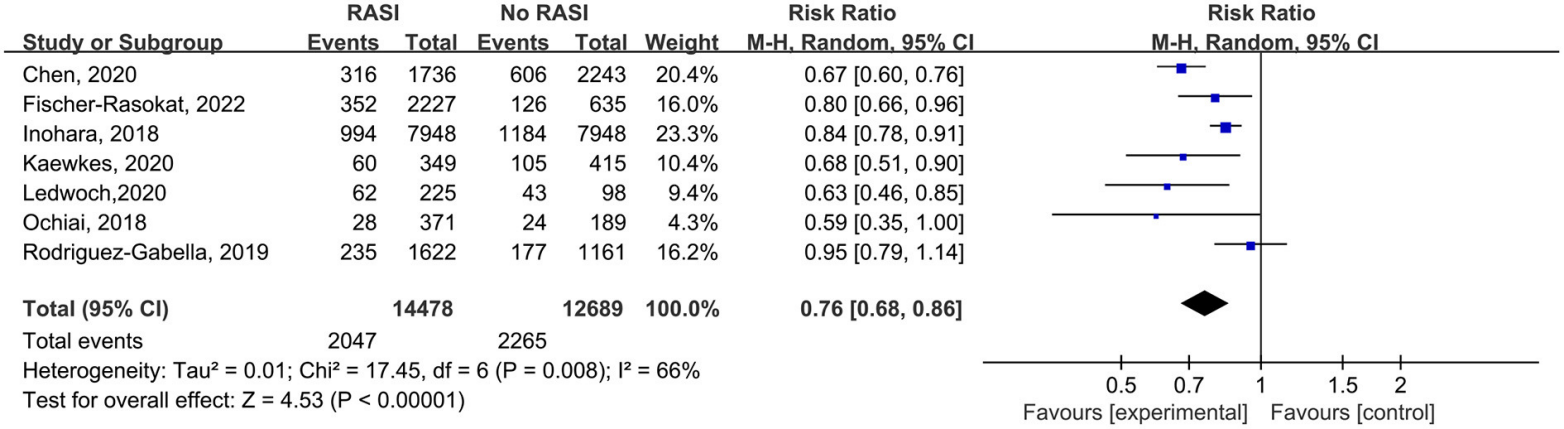
Forest plots of random-effects meta-analysis for the rate of all-cause mortality.

All studies reported the relationship between all-cause mortality and RAS inhibitors in patients after TAVR. There was a significantly lower rate of all-cause mortality in patients with RAS inhibitors than controls (RR = 0.76, 95%Cl = 0.68–0.86, *P* < 0.01, Figure 2). Patients with RAS inhibitors also had a lower rate of all-cause mortality after propensity matching (RR = 0.82, 95%Cl = 0.76–0.89, *P* < 0.01, Figure 4).

#### Secondary outcomes

**Figure 3:**
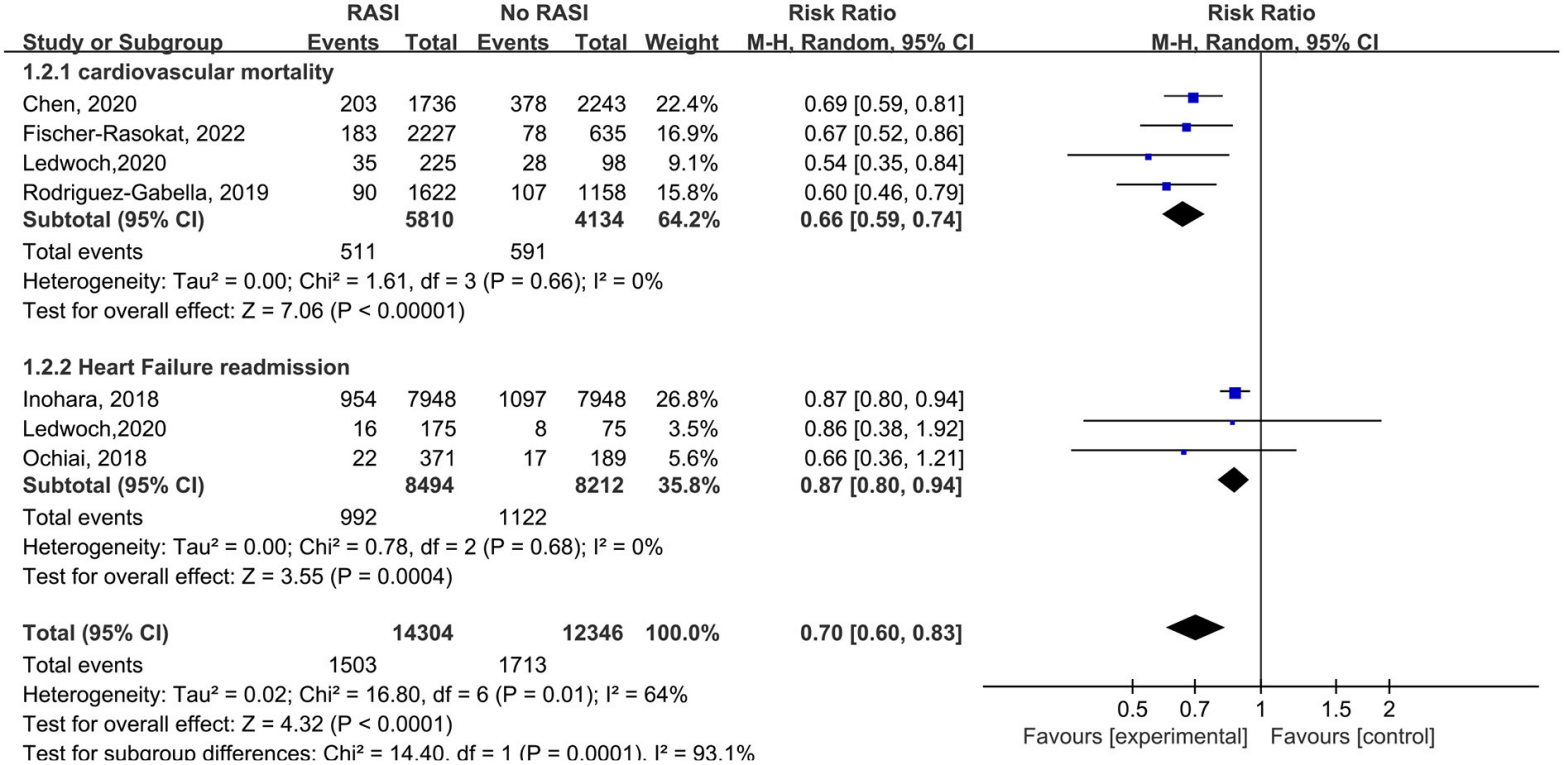
Forest plots of random-effects meta-analysis for the rates of cardiovascular death and heart failure readmission.

**Figure 4:**
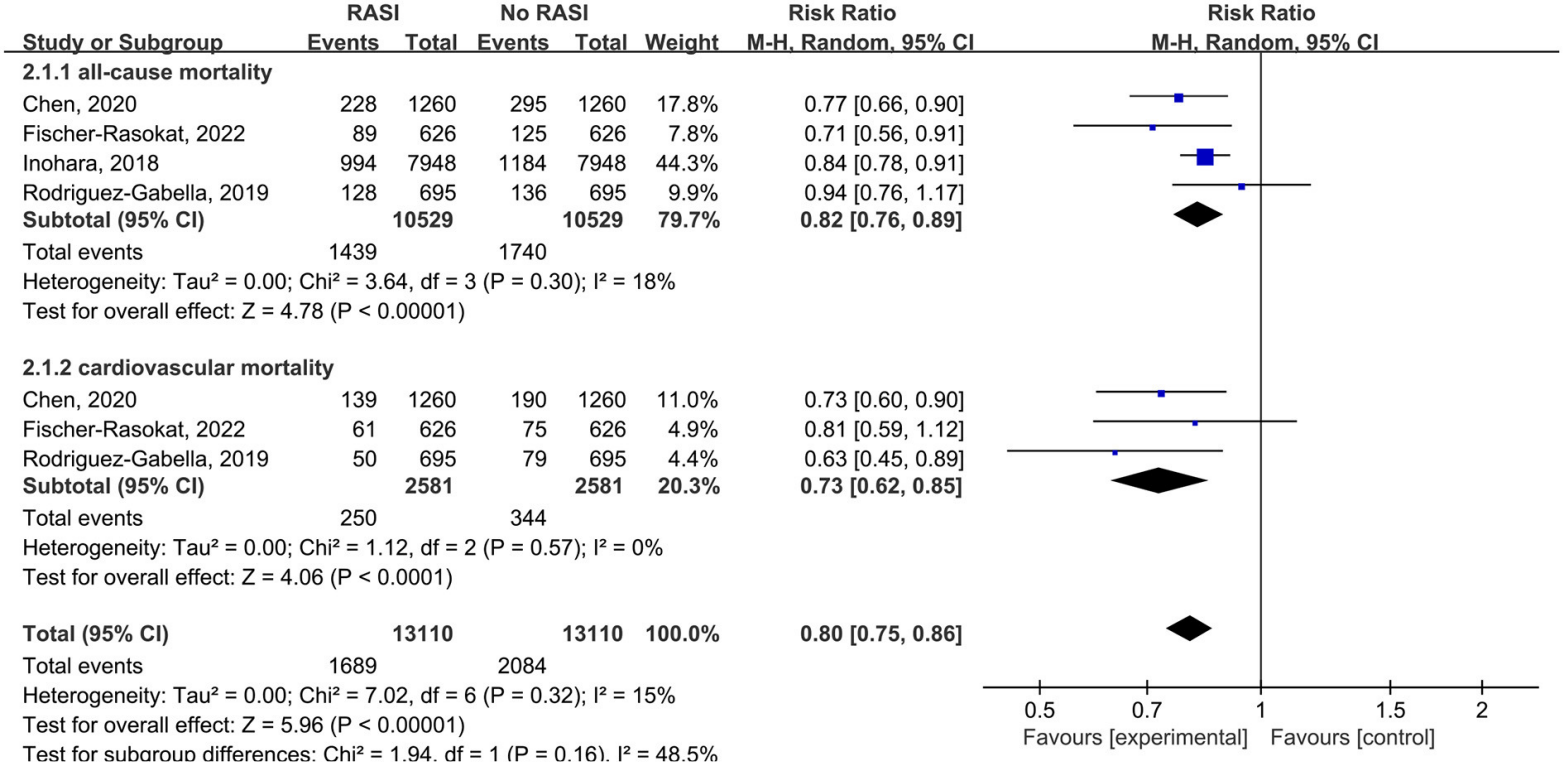
Forest plots of random-effects meta-analysis for the rates of all-cause mortality and cardiovascular death after propensity matching.

Four studies reported the relationship between cardiovascular death and RAS inhibitors in patients after TAVR. Patients treated with RAS inhibitors had a lower rate of cardiovascular death compared with controls (RR = 0.66, 95%Cl = 0.59–0.74, *P* < 0.01, Figure 3). Patients with RAS inhibitors also had a lower rate of cardiovascular death after propensity matching (RR = 0.73, 95%Cl = 0.62–0.85, *P* < 0.01, Figure 4).

Three studies reported the relationship between RAS inhibitors and HF readmission in patients after TAVR. Patients with RAS inhibitors had a lower HF readmission rate compared with controls (RR = 0.87, 95%Cl = 0.80–0.94, *P* < 0.01, Figure 3).

### Subgroup analysis and meta-regression analysis

**Figure 5:**
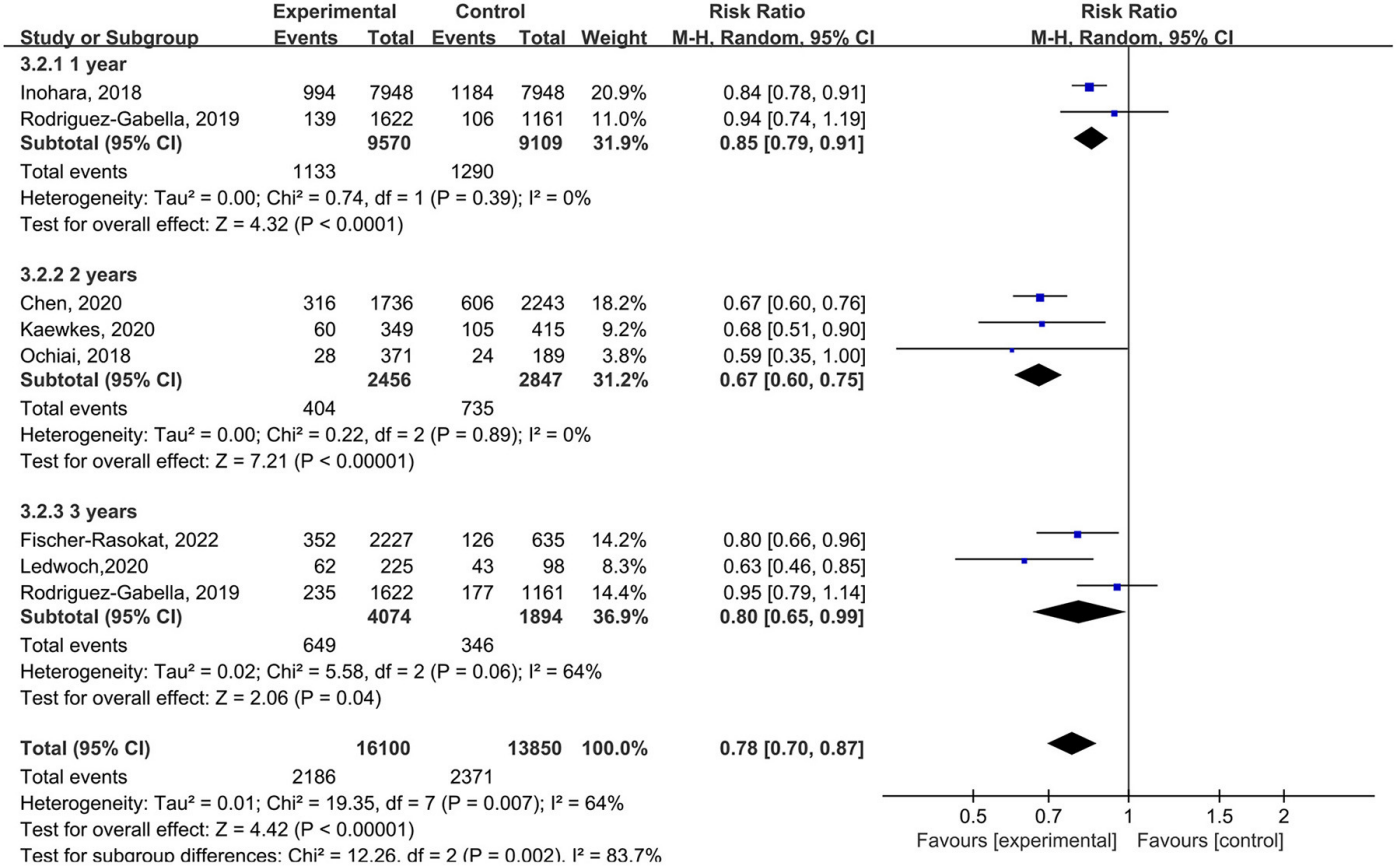
Forest plots of random-effects meta-analysis for the rate of all-cause mortality by subgroups of the follow-up period.

In subgroup analysis, for the follow-up period, the rate of all-cause mortality was lower in patients with RAS inhibitors compared with controls in 1 year (RR = 0.85, 95%Cl = 0.79–0.91, *P* < 0.01), 2 years (RR = 0.67, 95%Cl = 0.60–0.75, *P* < 0.01), 3 years (RR = 0.80, 95%Cl = 0.65–0.99, *P* = 0.04) (Figure 5).

**Figure 6:**
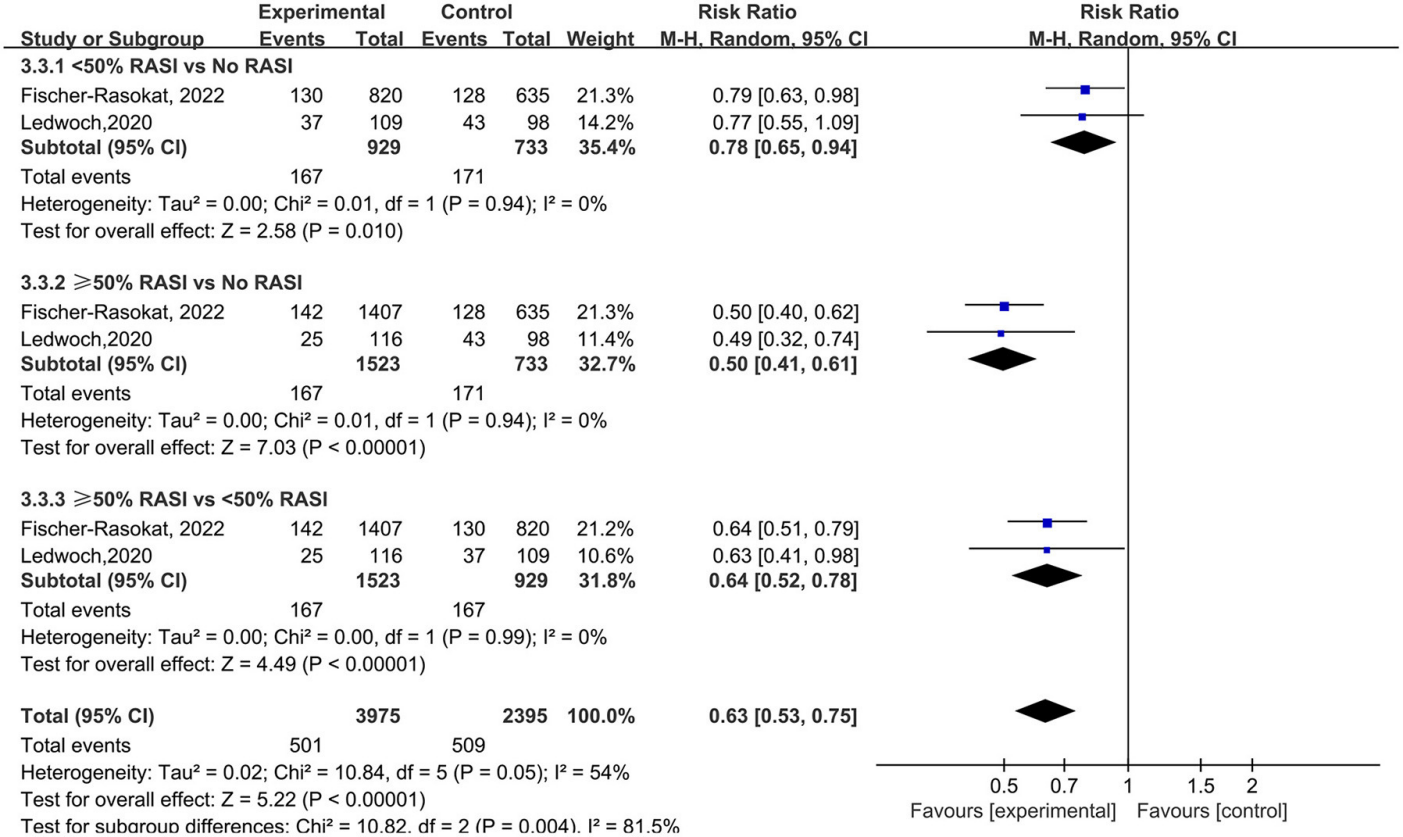
Forest plots of random-effects meta-analysis for the rate of all-cause mortality by subgroups of the dose of RAS inhibitors.

For the dose of RAS inhibitors, RAS inhibitors had a lower rate of all-cause mortality in patients with <50% (RR = 0.78, 95%Cl = 0.65–0.94, *P* = 0.01) and ≥50% (RR = 0.50, 95%Cl = 0.41–0.61, *P* < 0.01) target dose compared with control group. Then RAS inhibitors with ≥50% target dose had lower rate of all-cause mortality compared with <50% target dose (RR = 0.64, 95%Cl = 0.52–0.78, *P* < 0.01) (Figure 6).

The meta-regression analyses of included studies showed that age (*t* = −0.79, *P* = 0.465, Supplementary Table S4), sex (*t* = 1.21, *P* = 0.280, Supplementary Table S5) and baseline LVEF (*t* = 0.12, *P* = 0.910, Supplementary Table S6) did not have any modulating effect on all-cause mortality for the presence/absence of RAS inhibitors.

## Discussion

Our systematic review and meta-analysis investigated the impact of RAS inhibitors on clinical outcomes of patients after TAVR, and a total of 7 articles with a total of 32,585 patients (RAS inhibitor, *N* = 14,871; Controls, *N* = 17,714) were included. Our results demonstrated that the RAS inhibitors could lower the rates of all-cause mortality, cardiovascular death, and HF readmission. In subgroup analysis, the rate of all-cause mortality was lower in patients with RAS inhibitors compared with controls in 1 year, 2 years, and 3 years follow-up. Although RAS inhibitors had lower rate of all-cause mortality among both patients with <50% and ≥50% target dose compared with control group, RAS inhibitors with ≥50% target exhibited lower all-cause mortality. The meta-regression analyses of included studies found that age, sex and baseline LVEF did not have any modulating effect on all-cause mortality for the presence/absence of RAS inhibitors.

The association between the progression of HF and increased sympathetic nerve activity was well known, and RAS inhibitors may improve the clinical outcomes by reducing the sympathetic activity. RAS inhibitors had a positive effect on the regression of myocardial interstitial fibrosis and left ventricular hypertrophy. In the past, RAS inhibitors were not recommended to patients with AS since they may induce severe hypotension when fixed LV outflow obstruction existed, which was not proved by clinical evidence and was just based on theoretical risk. A previous systematic review and meta-analysis including eight trails (22–30) demonstrated that the prescription of RAS inhibitors for patients with aortic valve stenosis (AVS) may be safe, which was based on clinical practice. Our study showed that RAS inhibitors could lower the rates of all-cause mortality, cardiovascular death, and HF readmission in patients after TAVR, which may support the use of RAS inhibitors.

In included studies, three studies reported echocardiographic changes at follow-up. Rodriguez-Gabella et al found that patients with RAS inhibitors had larger regression of septal hypertrophy and a larger decrease in end-systolic and end-diastolic volumes (*p* < 0.001 for all). In Ledwoch's study, the results showed there was a larger reduction in LV mass index in patients with increasing RAS blockade doses significantly (*P* = 0.007). Ochiai et al demonstrated that there was greater LV mass index regression in patients with RAS inhibitors (−2 ± 25% vs. −9 ± 24%, *p* = 0.024) significantly. RAS inhibitors may enhance reverse LV remodeling and improve clinical outcomes and might be a suitable therapeutic option for patients after TAVR. For reduction of mortality and reverse remodeling, two studies have demonstrated that the impact of RAS inhibitors was dose-dependent. The results demonstrated that RAS inhibitors with ≥50% target dose were more effective than <50% target dose, and there was no significant difference between a full 100% target dose and a 50% target dose. It seems that RAS inhibitors with ≥50% target were sufficient to achieve full benefit for patients after TAVR.

In addition, RAS inhibitors were proved to be effective for patients after SAVR. Goel et al. demonstrated that RAS inhibitors could reduce the all-cause mortality in patients after SAVR due to severe AS (31). Magne et al showed that RAS inhibitors were associated with improved clinical outcomes for patients after SAVR (32). The results from a recent large real-world population-based study supported the use of RAS inhibitors for patients after SAVR due to AS (33). Three previous systematic reviews and meta-analysis (34–36) explored the impact of RAS inhibitors on patients after AVR (TAVR and/or SAVR), and demonstrated that RAS inhibitors could lower the all-cause mortality rate for patients after AVR. Our results were consistent with these studies and suggested the use of RAS inhibitors for patients after TAVR.

There were several limitations in our study. First, our study was based on the data from studies rather than patient-level data. Second, seven studies were included and more studies were needed. Due to the limited data from included studies, the pooled Kaplan-Meier curve with the overall population could not be reconstructed and it is unclear the preprocedural existence of RAS inhibitors. Finally, in the included studies, there were no randomized controlled trials. The potential confounders and bias may exist in included retrospective studies. Some ongoing RCTs including the RASTAVI trial (NCT03201185) and other trials (ChiCTR2100042266, ChiCTR 2100042266) will give us new evidence for this issue (37, 38).

In conclusion, our systematic reviews and meta-analysis demonstrated that RAS inhibitors could improve the clinical outcomes for patients after TAVR. Further large and high-quality trials should be conducted to support the use of RAS inhibitors for patients after TAVR.

## Data availability statement

The original contributions presented in the study are included in the article/Supplementary material, further inquiries can be directed to the corresponding authors.

## Author contributions

SW and JH: study design, research idea, and write the manuscript. YG and XL: data acquisition. SW and XL: data analysis and interpretation and statistical analysis. All authors contributed important work to this study and contributed to the article and approved the submitted version.

## Funding

This research was supported by the fellowship of the China Postdoctoral Science Foundation (Grant No. 2021M692802) and the Key Research and Development Program of Zhejiang Province (2020C03018).

## Conflict of interest

The authors declare that the research was conducted in the absence of any commercial or financial relationships that could be construed as a potential conflict of interest.

## Publisher's note

All claims expressed in this article are solely those of the authors and do not necessarily represent those of their affiliated organizations, or those of the publisher, the editors and the reviewers. Any product that may be evaluated in this article, or claim that may be made by its manufacturer, is not guaranteed or endorsed by the publisher.
