# Supplementary material for: The clinical outcomes of reni-angiotensin system inhibitors for patients after transcatheter aortic valve replacement: A systematic review and meta-analysis
Source: Front Cardiovasc Med. 2022 Aug 11;9:963731. doi: 10.3389/fcvm.2022.963731 (PMC9402980; doi:10.3389/fcvm.2022.963731)

**Supplementary materials**

**Table. S1: PRISMA checklist**

**Table. S2:** **The assessment of risk of bias with ROBINS-I tool**

**Table. S3: The types of the RAS inhibitors pre study**

**Table. S4: Meta-regression of age for all-cause mortality**

**Table. S5: Meta-regression of sex for all-cause mortality**

**Table. S6: Meta-regression of Baseline LVEF for all-cause mortality**

**Table. S1: PRISMA checklist**

**Table. S2:** **The assessment of risk of bias with ROBINS-I tool**

**Table. S3: The types of the RAS inhibitors pre study**

| **Study** | **Distribution of RAS inhibitor** |
| --- | --- |
| Chen, 2020 | ACEI (60.9%) or ARB (36.6%) |
| Fischer-Rasokat, 2022 | ACEI (60.9%) or ARB (39.1%) |
| Rodriguez-Gabella, 2019 | ACEI (48.6%) or ARB (40%) or Eplerenone/Spironolactone (3.3%) or Sacubitril/Valsartan (0.49%) or Multiple (7.6%) |
| Kaewkes, 2020 | ACEI (41%) or ARB (48%) or Sacubitril/Valsartan (0.5%) or aldosterone antagonist (6%) or Combination (4%) |
| Inohara, 2018 | ACEI (33%) or ARB (17.7%) or both (0.76%) |
| Ledwoch,2020 | ACEI 66.7% (Ramipril, Enalapril, Captopril, Lisinopril, Perindopril); or ARB 33.3% (Valsartan, Candesartan, Losartan, Olmesartan, Telmisartan, Eprosartan) |
| Ochiai, 2018 | ACEI or ARB |
|  |  |
| ACEI: Angiotensin converting enzyme inhibitors; ARB: Angiotensin receptor blocker | |

**Table. S4: Meta-regression of age for all-cause mortality**

**
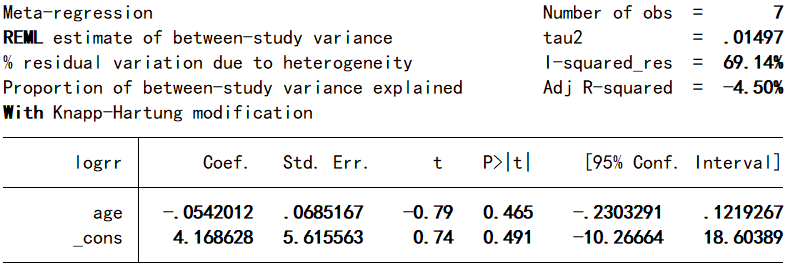
**

**Table. S5: Meta-regression of sex for all-cause mortality**

**
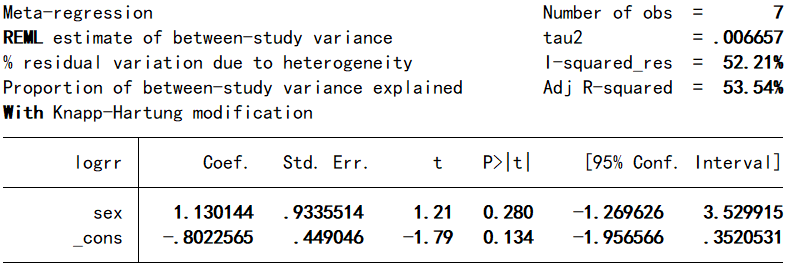
**

**Table. S6: Meta-regression of Baseline LVEF for all-cause mortality**


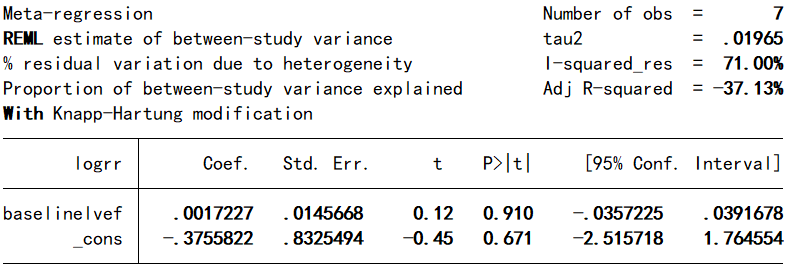

Supplement: Supplementary file 1 [file Data_Sheet_1.docx]
